# Dietary inflammatory index in patients with chronic kidney disease under conservative treatment at a university hospital in Rio de Janeiro, Brazil

**DOI:** 10.1590/2175-8239-JBN-2025-0350en

**Published:** 2026-08-17

**Authors:** Karine Campos Ladeira, Amália Almeida Bastos, Raquel Martins Martinez, Tatiana Pereira de Paula

**Affiliations:** 1 Universidade Federal do Rio de Janeiro, Instituto de Nutrição Josué de Castro, Programa de Pós-Graduação em Nutrição Clínica, Rio de Janeiro, RJ, Brazil.; 2 Universidade de São Paulo, Programa de Pós-Graduação em Nutrição em Saúde Pública, São Paulo, SP, Brazil.; 3 Universidade Federal do Estado do Rio de Janeiro, Programa de Pós-Graduação em Alimentação e Nutrição, Rio de Janeiro, RJ, Brazil.

**Keywords:** Renal Insufficiency, Chronic, Inflammation, Diet, Heart Disease Risk Factors, Nutritional Status.

## Abstract

**Introduction:**

Chronic kidney disease (CKD) is associated with a chronic low-grade inflammatory state, which may be influenced by dietary patterns. The Dietary Inflammatory Index (DII) is a validated tool used to estimate the inflammatory potential of the diet.

**Objective:**

To evaluate the dietary inflammatory potential in patients with CKD undergoing conservative treatment and its association with anthropometric and cardiovascular risk markers.

**Methods:**

A cross-sectional observational study was conducted in adult patients diagnosed with stage 3–5 CKD under conservative management. Dietary intake was assessed using two non-consecutive 24-hour dietary recalls, and DII scores were calculated based on 36 dietary parameters. Anthropometric and cardiovascular risk indicators were evaluated, including body mass index (BMI), triceps skinfold thickness (TSFT), arm muscle area (AMA), waist-to-height ratio (WHtR), Castelli indices, and the conicity index (CI). Multivariate linear regression models were adjusted for age, sex, and estimated glomerular filtration rate (eGFR).

**Results:**

A total of 51 patients were included, with a median age of 71 years (IQR, 56–80), and 56.9% were female. The median DII score was 2.90 (IQR, 2.29–3.74), indicating a predominantly pro-inflammatory dietary profile. Higher DII scores were associated with higher conicity index values (β = 0.368; *p* = 0.010). No statistically significant associations were observed between the DII and other anthropometric, biochemical, or cardiovascular risk markers.

**Conclusion:**

Patients with CKD under conservative treatment exhibited predominantly pro-inflammatory dietary patterns. Higher DII scores were associated with greater central adiposity.

## INTRODUCTION

Over 850 million people worldwide suffer from some form of kidney disease, with chronic kidney disease (CKD) responsible for over one million deaths each year. It is estimated that population aging and growth will lead to a significant increase in the prevalence of CKD in the coming decades, particularly in low and middle-income countries, where large proportions of the population lack access to diagnosis, prevention, and treatment^1^.

CKD is characterized by a progressive and irreversible loss of kidney function over a period of at least three months. This decline can be detected by imaging, blood, and urine tests^1^. The progression of the disease leads to a decrease in the urinary excretion of ions, electrolytes, and especially the nitrogenous products of protein metabolism and dietary protein intake. Consequently, low-grade metabolic acidosis ensues, accompanied by clinical and nutritional complications, thereby increasing the risk of morbidity and mortality^2,3^.

The mechanisms underlying CKD generate a chronic and persistent inflammatory process with multifactorial causes. These include increased production of pro-inflammatory cytokines, oxidative stress, acidosis, chronic and recurrent infections, altered adipose tissue metabolism, and intestinal dysbiosis. These factors result in repeated vascular endothelial damage, scarring, and fibrosis^2,3^.

The rigorous control of modifiable risk factors is aimed at reducing the progression of the disease. The following factors must be considered: systemic arterial hypertension (SAH), diabetes and glycemic control, dyslipidemia, obesity, smoking, and dietary habits^1,4^.

Dietary patterns rich in antioxidants, anti-inflammatory compounds, and nutritionally balanced patterns of consumption, such as those that emphasize the ingestion of plant-based foods, as observed in the Mediterranean diet, the Dietary Approaches to Stop Hypertension (DASH), as well as vegetarian and vegan diets, have been demonstrated to be efficacious in the attenuation of inflammation in CKD^5^. In addition to the nutritional composition of a healthy diet, the presence of phytochemicals confers substantial anti-inflammatory effects. Bioactive compounds, such as resveratrol, allicin, cinnamaldehyde, quercetin, proanthocyanidin, catechin, curcumin, epigallocatechin-3-gallate, fisetin, sulforaphane, and chlorogenic acids, have been shown to modulate NRF2 expression, suppress NLRP3 expression, and regulate the inflammatory and pro-oxidative characteristics in patients with CKD^5^.

The Dietary Inflammatory Index (DII) is a validated tool that has demonstrated good capacity for assessing the inflammatory potential of a diet. It was developed based on the association between the inflammatory potential of the diet and serum concentrations of C-reactive protein (CRP). The DII is estimated using weighted scores based on scientific evidence, considering dietary components with anti-inflammatory and pro-inflammatory properties. The higher and more positive the score, the more pro-inflammatory the diet, while lower and more negative values are associated with an anti-inflammatory diet^6^.

Some authors have demonstrated an important relationship between the DII and chronic kidney disease. Mazidi et al.^7^ investigated the association between the DII and the prevalence of CKD in American adults participating in the National Health and Nutrition Examination Survey (NHANES) with measured data on renal function markers from 2005 to 2012, including 21,649 participants, of whom 1,634 (6.8%) had CKD. Among other findings, the authors concluded that a pro-inflammatory diet is associated with decreased renal function and a high prevalence of CKD. In a meta-analysis, Kachouei et al.^8^ demonstrated a significant positive association between DII and the likelihood of CKD and low glomerular filtration rate (GFR), although the results regarding the correlation between DII and serum biomarkers of renal function remain inconclusive. Hence, studies evaluating the use of this index in CKD are still scarce, requiring more comprehensive data collection in other regions of the world to validate the association.

Since CKD is associated with a chronic inflammatory condition, a high score on the DII may also be associated with unfavorable clinical outcomes. Therefore, the primary objective of this study was to investigate the association between the DII and anthropometric and atherogenic markers in patients with chronic kidney disease undergoing conservative treatment. Secondary objectives included describing the dietary inflammatory profile of the study population and comparing dietary, clinical, and sociodemographic characteristics according to DII categories.

## METHODS

### Study design

For this cross-sectional study, a convenience sample of patients with a clinical diagnosis of CKD undergoing conservative treatment or receiving follow-up care at the Nephrology Outpatient Clinic of the *Hospital Universitário Clementino Fraga Filho* (HUCFF/Rio de Janeiro, RJ, Brazil) was considered, without prior consultation at the nutrition outpatient clinic. No formal sample size calculation was performed, and the study should be considered exploratory.

The following criteria were used to determine eligibility: age over 18 years, any sex, estimated glomerular filtration rate (eGFR) less than or equal to 60 mL/min, absence of autoimmune diseases (e.g., glomerulopathies) and malignant neoplasms, and no history of previous kidney transplantation or dialysis therapy. Patients who declined to participate in a second 24-hour dietary recall were excluded from the study.

The study was submitted to and approved by the HUCFF Research Ethics Committee, approval No. 21.941-913, in accordance with the recommendations of Resolution No. 466/12 of the National Health Council. Participation in the study was voluntary, and all participants were able to read and sign the Informed Consent Form.

### Sociodemographic profile

Information was collected on sociodemographic characteristics (age, education, *per capita* income, race), length of treatment at HUCFF, physical exercise, smoking, alcohol consumption, biochemical tests, anthropometric measurements, and data on food consumption. Information regarding chronic diseases and medication use was obtained through structured self-report during face-to-face interviews conducted by the researcher and subsequently confirmed through review of patients’ medical records. All data were collected by a single nutritionist researcher.

Current smokers are defined as those who have smoked 100 or more cigarettes during their lifetime and who continue to smoke; former smokers are defined as those who have smoked at least 100 cigarettes during their lifetime but have stopped smoking, regardless of how long ago they stopped^9^. Alcohol consumers were defined as those who consumed four or more drinks for women and five or more drinks for men on a single occasion in the last 30 days. One drink is defined as a standard measure of a distilled beverage, a glass of wine, or a can of beer. Former drinkers were defined as those who consumed alcohol in the past but did not drink in the current year^4^. The level of education was assessed in two groups: low education (incomplete or complete elementary school or incomplete high school) and high education (complete high school, incomplete or complete higher education). The classification of *per capita* family income was determined based on a comparison with the Brazilian minimum wage in 2023, with income levels categorized as equal to or less than one minimum wage or greater than one minimum wage. In terms of physical activity, individuals were considered active if they engaged in a minimum of 150 to 300 minutes of moderate-intensity aerobic physical activity, or a minimum of 75 to 150 minutes of vigorous-intensity aerobic physical activity, or an equivalent combination of moderate and vigorous-intensity physical activities throughout the week. Aerobic activities were categorized as endurance activities that enhance cardiorespiratory fitness, including walking, running, swimming, and cycling^10^.

### Nutritional profile

Anthropometric measurements of body weight, height, arm circumference (AC), waist circumference (WC), and triceps skinfold thickness (TSFT) were collected according to established protocols^11,12^. Body mass was measured using a digital scale (FILIZOLA® PL 180) with a maximum capacity of 150 kg and an accuracy of 0.1 kg. The height of the participants was measured in meters using a wall-mounted stadiometer (TONELLI®) with an accuracy of 0.1 cm. Circumference and skinfold measurements were obtained using the following instruments: a flexible steel anthropometric tape measure and a scientific adipometer (CESCORF®).

The estimation of body mass index (BMI) was based on body weight and height measurements. Nutritional diagnosis was based on BMI values: normal weight (BMI = 18.5–24.9 kg/m^2^), overweight (BMI = 25–29.9 kg/m^2^), grade I obesity (BMI = 30–34.9 kg/m^2^), and grade II obesity (BMI = 35–39.9 kg/m^2^). In the case of older adults, the following BMI categories were used: underweight (BMI < 23 kg/m^2^), adequate weight (23 < BMI < 28 kg/m^2^), overweight (28 < BMI < 30 kg/m^2^), and obesity (BMI > 30 kg/m^2^)^12,13^.

To assess muscle mass, the arm muscle area (AMA) was calculated: AMA = [AC – (TSFT × π)]^2^ ÷ (4 × π). The values were compared with those recommended by Frisancho^14^ according to age and sex, with individuals with percentile values below 15 being classified as having lean mass depletion and those with percentile values below 5 being classified as having severe depletion.

### Biochemical profile and renal function

Laboratory test results were obtained from the HUCFF computerized patient care system, dated no more than two months prior to data collection. The serum parameters analyzed were urea, creatinine, glucose, glycated hemoglobin (HbA1c), hemoglobin, albumin, total cholesterol, high-density lipoprotein cholesterol (HDL-c), low-density lipoprotein cholesterol (LDL-c), triglycerides, phosphorus, potassium, calcium, parathyroid hormone (PTH), and vitamin D. Renal function was assessed by calculating eGFR using the CKD Epidemiology Collaboration (CKD-EPI) equation, recommended by the National Kidney Foundation guidelines for the Kidney Disease Outcomes Quality Initiative^1^.

### Indicators of abdominal adiposity and atherogenic risk

Central adiposity was assessed by the waist-to-height ratio (WHtR), with a cutoff value of 0.55 determined by Lin et al.^15^ to indicate high central adiposity. The assessment of abdominal fat is predicated on the calculation of the conicity index (CI), which was determined by means of the following mathematical equation: CI = WC (m)/[0.109 × (√P(kg)/A(m))]^16^. The values proposed by Pitanga and Lessa^17^ were used for the classification of cardiovascular risk, equivalent to ≥ 1.18 for women and ≥ 1.25 for men.

The Castelli I (ratio between total cholesterol and HDL-c) and II (ratio between LDL-c and HDL-c) indices were used to assess atherogenic risk. The following reference values were considered: Castelli Index I < 4.0 for women and < 5.0 for men, and Castelli Index II < 3.0 for women and < 3.5 for men^18,19^.

### Food consumption

Food intake was assessed using a 24-hour recall questionnaire (24HR), employing the multiple-pass method validated by Conway et al.^20^, which consists of five steps: 1) uninterrupted listing of foods and beverages consumed; 2) questioning about food groups that are frequently consumed; 3) recording the time and occasion of food consumption; 4) detailing the quantities and descriptions of foods consumed, aided by a photo album; 5) final review of the information provided. Two questionnaires were administered on different, non-consecutive days, with the objective of achieving an adequate proportion of weekdays and weekends within the study population. The first questionnaire was administered in person, whereas the second was conducted by telephone.

The portions of food consumed were described in household measures and subsequently converted into mass and volume using the Table for Evaluating Food Consumption in Household Measures^21^. Macronutrients, minerals, and energy were estimated based on the Brazilian Food Composition Table^22^. The flavonoid and carotenoid contents of foods were determined using supplementary tables in the same database^23,24^. The *Phenol Explorer* database was also used^25^. The caffeine values for foods correspond to the estimates proposed by Rocha et al.^26^. The quantities of garlic and onion were estimated for each 100 g of preparation: for rice and meat, 1 g of garlic and 4 g of onion; for beans, 0.2 g of garlic and 4 g of onion. For seasonings, per 100 g of preparation, 0.35 g of oregano, 0.74 g of black pepper, and 0.4 g of rosemary were considered when used.

The nutrient and food data from the two recall surveys were used to estimate each individual’s usual intake. The distribution of consumption was corrected for intra-individual variability using the Multiple Source Method (MSM) program, which calculates the individual intake of each nutrient and food analyzed.

### Dietary inflammatory index (DII)

The method described by Shivappa et al.^27^ was employed to calculate the DII, taking into account the variation between pro-inflammatory (assigned positive scores) and anti-inflammatory (assigned negative scores) components. To obtain the DII score, 36 dietary parameters were considered: folic acid, monounsaturated fatty acids, polyunsaturated fatty acids, β-carotene, carbohydrates, proteins, cholesterol, energy, fiber, flavonols, flavones, flavanones, flavan-3-ols, anthocyanidins, saturated fat, total fat, trans fat, vitamins B1, B2, B3, B6, B12, A, D, C, and E, magnesium, zinc, iron, selenium, garlic, onion, oregano, pepper, rosemary, and caffeine. The parameters eugenol, alcohol, ginger, turmeric, green tea, and black tea were excluded from the DII calculation because no intake was reported for these items in the study population. This approach is consistent with previous studies applying the DII when complete dietary data are unavailable^2,7^. The presence of isoflavones and turmeric was observed in the diet of only one patient and, therefore, was not considered part of the usual dietary intake of the study population.

The score for each parameter was calculated and then summed to obtain an individual DII score. Subsequently, the scores calculated for all dietary parameters were summed to obtain the total DII score for each individual. To minimize differences in energy intake between individuals, the DII was adjusted for energy by converting all foods to 1,000 kcal.

### Statistical analyses

Statistical analyses were performed using Statistical Package for the Social Sciences® software, version 22 for Windows (SPSS® Inc., Chicago, IL). The Shapiro-Wilk test was used to assess the distribution of variables. Continuous variables with a normal distribution were described as means and standard deviations (SD), while those with a nonparametric distribution were described as medians and interquartile ranges (IQR). Categorical variables were described using absolute (n) and relative (%) frequencies.

DII categorization based on the median was used only for exploratory descriptive comparisons, while the DII was maintained as a continuous variable in regression analyses. Multicollinearity was assessed through correlation analysis between independent variables, and only variables not intrinsically related to the DII calculation were included in multivariate models.

To evaluate the association between dietary inflammatory potential and anthropometric cardiovascular risk markers, multivariate linear regression analysis was performed with the continuous CI as the dependent variable and the DII score as the independent predictor of interest. The model was adjusted for age, sex, and eGFR. Statistical significance was set at *p* < 0.05.

## RESULTS

Of the total of 61 patients with CKD, 10 patients were excluded due to the absence of dietary intake data. The median DII was 2.9029 (IQR, 2.29–3.74). Sociodemographic and lifestyle characteristics stratified according to low and high DII scores are presented in Table 1. No significant differences were observed between DII groups for age, sex, physical activity, smoking status, alcohol consumption, primary diagnosis of CKD, or income (*p* > 0.05), whereas educational level differed significantly between groups (*p* = 0.039).

**Table 1:** Sociodemographic characteristics of CKD patients undergoing conservative treatment at a university hospital in Rio de Janeiro, Brazil, stratified by DII score, based on the median value observed in this population.

Characteristics	(DII < 2.9029)	(DII > 2.9029)	*p*
**Age (years)**			
*Median (IQR)*	70 (39–88)	71.5 (31–86)	0.116
**Sex**			
Male	52% (n = 13)	65.4% (n = 17)	0.157
Female	48% (n = 12)	34.6% (n = 9)
**Physical activity**			
Physically active	8% (n = 2)	0% (n = 0)	0.216
Physically inactive	92% (n = 23)	100% (n = 26)
**Smoking status**			
Never smoker	82.4% (n = 20)	82.4% (n = 22)	0.187
Former smoker	17.6% (n = 5)	17.6% (n = 4)
Current smoker	0% (n = 0)	0% (n = 0)
**Alcohol consumption**			
Non-drinker	86.3% (n = 20)	92.3% (n = 24)
Former drinker	13.7% (n = 5)	7.7% (n = 2)	0.163
Current drinker	0% (n = 0)	0% (n = 0)
**Educational level**			
Low educational level	66.7% (n = 17)	65.4% (n = 17)	0.039
High educational level	33.3% (n = 8)	34.6% (n = 9)
**Primary diagnosis of CKD**			
Hypertension	72% (n = 18)	53.8% (n = 14)	0.0862
Type 2 diabetes mellitus	12% (n = 3)	15.4% (n = 4)
Other causes	16% (n = 4)	30.8% (n = 8)
***Per capita* family income according to minimum wage (MW)**			
≤ 1 minimum wage	68% (n = 17)	73.1% (n = 19)	0.688
> 1 minimum wage	32% (n = 8)	26.9% (n = 7)

Notes – Age presented as median and interquartile range (IQR); all other variables presented as frequency (%). Physical activity defined as engaging in at least 150–300 minutes of moderate-intensity aerobic physical activity, or at least 75–150 minutes of vigorous-intensity aerobic physical activity, or an equivalent combination of moderate and vigorous-intensity physical activity throughout the week. Former smokers defined as individuals who had smoked at least 100 cigarettes during their lifetime but had quit smoking. Former drinkers defined as individuals who had consumed alcohol in the past but had not consumed alcohol during the current year. High educational level defined as completion of high school, incomplete higher education, or completed higher education. Family income was classified according to the Brazilian minimum wage for the year 2023.

All selected patients reported using antihypertensive medications, with a notable proportion (n = 34) also using lipid-lowering medications, 41.18% (n = 21) using hypoglycemic medications, and 15.69% (n = 8) using cholecalciferol. According to the primary diagnosis of CKD, 32 individuals had SAH (62.7%), followed by type 2 diabetes mellitus (13.7%), polycystic kidney disease (7.8%), nephrolithiasis (5.9%), urinary tract infection (3.9%), pyelonephritis (2%), solitary kidney (2%), and type 1 diabetes mellitus (2%). The duration of CKD treatment at the HUCFF nephrology outpatient clinic was 2.67 ± 1.7 years.

Regarding biochemical parameters, the values of albumin (4 ± 0.56 g/dL), triglycerides (136 ± 69.27 mg/dL), total cholesterol (180 mg/dL; IQR, 151–203), LDL-c (101.7 ± 41.52 mg/dL), potassium (4.82 ± 0.62 mEq/L), phosphorus (4.04 ± 0.82 mg/dL), and calcium (9.24 ± 0.63 mg/dL) were within the normal range. The median HbA1c was elevated (6.3% (5.6–7.7)). The other altered parameters were consistent with the presence of CKD.

The associations between the DII and the dietary components included in the index calculation are presented in Table 2.

**Table 2:** Comparison of nutrient intake among patients with CKD stratified according to the median DII, treated at a university hospital in Rio de Janeiro, Brazil.

Parameter	(DII < 2.9029)	(DII > 2.9029)	*p*-value
Calories (kcal)	1512.66 ± 197.851	1567.17 ± 306.48	0.456
Protein (g)	78.47 ± 9.66	74.41 ± 14.76	0.254
Carbohydrate (g)	212.17 ± 31.98	198.48 ± 33.69	0.143
Lipids (g)	48.16 ± 4.69	51.22 ± 7.14	0.790
Fiber (g)	31.06 ± 5.17	21.67 ± 6.51	0.000
Cholesterol (mg)	274.67 ± 89.30	268.69 ± 88.22	0.811
Saturated fat (g)	19.56 ± 0.40	19.40 ± 0.437	0.185
Monounsaturated fat (g)	14.54 ± 1.83	15.63 ± 2.67	0.094
Polyunsaturated fat (g)	6.88 ± 2.21	8.71 ± 2.74	0.011
Trans fat (g)	1.22 ± 0.44	1.57 ± 0.57	0.019
Calcium (mg)	645.13 ± 109.13	615.69 ± 155.93	0.441
Iron (mg)	11.01 ± 1.56	10.48 ± 2.39	0.355
Magnesium (mg)	257.56 ± 35.28	219.25 ± 39.35	0.001
Zinc (mg)	11.48 ± 1.56	10.35 ± 3.04	0.106
Flavonols (mg)	0.55 (0.1–3.40)	0.40 (0.05–1.30)	0.297
Garlic (g)	4.20 (2.95–4.20)	3.20 (2.20–4.20)	0.075
Onion (g)	20.00 (12.10–20.00)	16.00 (12.10–20.00)	0.160
Caffeine (mg)	75.15 (10.57–137.50)	67.71 (11.60–116.50)	0.901
β-carotene (µg)	699.60 (39.4–2577.5)	442.20 (253.90–442.20)	0.304
Black pepper (g)	0.40 (0.35–0.60)	0.40 (0.20–0.55)	0.603
Selenium (mcg)	40.65 (30.67–50.55)	38.30 (30.25–46.60)	0.977
Vitamin A RE (mcg)	389.53 ± 85.18	386.41 ± 82.14	0.895
Vitamin D (mcg)	1.25 (0.45–2.32)	1.70 (0.20–3.25)	0.962
Vitamin C (mg)	108.80 (52.80–150.50)	49.80 (33.90–85.150)	0.001
Vitamin B1 (mg)	1.03 ± 0.16	1.03 ± 0.27	0.984
Vitamin B2 (mg)	1.50 ± 0.46	1.60 ± 0.55	0.483
Vitamin B3 (mg)	20.30 ± 4.80	20.94 ± 5.67	0.665
Vitamin B6 (mg)	0.80 (0.50–1.00)	0.80 (0.60–1.00)	0.614
Vitamin B12 (mcg)	3.20 (2.70–4.20)	3.20 (2.40–4.90)	0.977
Vitamin E (mg)	4.53 ± 0.78	4.62 ± 0.85	0.697
Vitamin B9 (mcg)	432.15 (399.20–488.00)	348.00 (290.50–413.30)	0.000
Sodium (mg)	1432.77 ± 200.33	1596.95 ± 260.16	0.015
Copper (mg)	1.81 ± 0.33	1.33 ± 0.41	0.000
Phosphorus (mg)	1147.21 ± 177.80	1094.77 ± 216.73	0.349
Potassium (mg)	2465.36 ± 278.35	2215.44 ± 298.08	0.003

Abbreviation – Vitamin A RE: Vitamin A retinol equivalent.

Table 3 presents the nutritional profile and cardiovascular risk factors of patients with chronic kidney disease stratified according to the median DII. Among adults, eutrophic individuals predominated in both DII groups, whereas overweight was more frequent among older adults, particularly in the group with higher DII scores. Regarding AMA, most participants presented values within the adequate range (percentile ≥ 16 and ≤ 85) in both groups.

**Table 3:** Nutritional profile and cardiovascular risk factors of CKD patients treated at a university hospital in Rio de Janeiro, Brazil, stratified by DII score, based on the median value observed for this population.

Parameters	(DII < 2.9029)	(DII > 2.9029)	*p*
**BMI – adults (n = 15)**			
< 18.50 kg/m^2^	0% (n = 0)	6.67% (n = 1)	0.645
≥ 18.50 kg/m^2^ – ≤ 24.90 kg/m^2^	26.67% (n = 4)	26.67% (n = 4)
≥ 25.00 kg/m^2^ – ≤ 29.90 kg/m^2^	6.67% (n = 1)	6.67% (n = 1)
≥ 30.00 kg/m^2^ – ≤ 34.90 kg/m^2^	0% (n = 0)	13.33% (n = 2)
≥ 34.90 kg/m^2^ – ≤ 39.90 kg/m^2^	6.67% (n = 1)	6.67% (n = 1)
**BMI – older adults (n = 36)**			
< 23.00 kg/m^2^	16.67% (n = 6)	8.33% (n = 3)	0.619
≥ 23.00 kg/m^2^ – ≤ 28.00 kg/m^2^	16.67% (n = 6)	16.67% (n = 6)
> 28.00 kg/m^2^	19.44% (n = 7)	22.22% (n = 8)
**AMA (n = 51)**			
Percentile < 5	3.92% (n = 2)	0% (n = 0)	0.453
Percentile ≥ 5 – ≤ 15	5.88% (n = 3)	7.84% (n = 4)
Percentile ≥ 16 – ≤ 85	33.33% (n = 17)	33.33% (n = 17)
Percentile ≥ 95	5.88% (n = 3)	9.80% (n = 5)
**WHtR (n = 51)**			
≤ 0.55	29.41% (n = 15)	19.61% (n = 10)	0.473
≥ 0.55	21.57% (n = 11)	29.41% (n = 15)
**CI – female (n = 29)**			
≤ 1.18	20.69% (n = 6)	20.69% (n = 6)	0.341
≥ 1.18	20.69% (n = 6)	37.93% (n = 11)
**CI – male (n = 22)**			
≤ 1.25	36.36% (n = 8)	22.73% (n = 5)	0.082
≥ 1.25	9.09% (n = 2)	31.82% (n = 7)
**Castelli Index I – female (n = 28)**			
≤ 4.00	25.00% (n = 7)	17.86% (n = 5)	0.299
≥ 4.00	42.86% (n = 12)	14.29% (n = 4)
**Castelli Index I – male (n = 18)**			
≤ 5.00	50.00% (n = 9)	5.56% (n = 1)	0.556
≥ 5.00	44.44% (n = 8)	0% (n = 0)
**Castelli Index II – female (n = 25)**			
≤ 3.00	36.00% (n = 9)	8.00% (n = 2)	0.305
≥ 3.00	36.00% (n = 9)	20.00% (n = 5)
**Castelli Index II – male (n = 21)**			
≤ 3.50	52.38% (n = 11)	0% (n = 0)	0.476
≥ 3.50	42.86% (n = 9)	4.76% (n = 1)

Abbreviations – BMI: body mass index; AMA: arm muscle area; WHtR: waist-to-height ratio; CI: conicity index.

Concerning central adiposity indicators, individuals with higher DII scores showed a greater frequency of elevated WHtR and higher proportions of elevated CI in both women and men. Among women, 37.93% of participants with DII scores above the median presented elevated CI values, while among men this proportion was 31.82%.

For the atherogenic indices, a predominance of elevated Castelli index I values was observed among women with DII scores below the median. Among men, most participants presented normal values for Castelli indices I and II. No statistically significant differences were observed between groups for the evaluated variables.

In the context of cardiovascular risk assessment, Table 4 demonstrates a positive correlation between the DII and the CI (*r* = 0.333; *p* = 0.017), indicating an association between higher dietary inflammatory potential and greater central adiposity. No statistically significant correlations were observed between DII and the other evaluated cardiovascular risk markers.

**Table 4:** Correlation between DII and cardiovascular risk variables in CKD patients treated at a university hospital in Rio de Janeiro, Brazil.

Cardiovascular risk	*r*	*p*-value
CI	0.333	0.017
Castelli Index I	0.239	0.091
Castelli Index II	0.009	0.949
WHtR	0.247	0.081

Abbreviations – WHtR: waist-to-height ratio; CI: conicity index; *r*: correlation coefficient.

In multivariate linear regression analysis using the continuous CI as the dependent variable and adjusted for age, sex, and eGFR, higher DII scores were positively associated with higher CI values (*B* = 0.043; standardized β = 0.368; *p* = 0.010) (Table 5). No statistically significant associations were observed for age, sex, or eGFR.

**Table 5:** Multivariate linear regression analysis of factors associated with the continuous conicity index in CKD patients treated at a university hospital in Rio de Janeiro, Brazil.

Variables	Adjusted effects			
	*B*	Standardized β	*t*	*p*-value
DII score	0.043	0.368	2.669	0.010
Age (years)	0.001	0.143	0.892	0.377
Sex	0.000	0.001	0.008	0.993
eGFR	-0.001	-0.106	-0.668	0.508

Notes – Model adjusted for age, sex, and eGFR, presenting unstandardized coefficients (*B*) and standardized coefficients (β). Statistical significance level set at *p* < 0.05. Model fit: R^2^ = 0.156; adjusted R^2^ = 0.083; *F* = 2.129; *p* = 0.092.

## DISCUSSION

The present study demonstrated that patients with CKD undergoing conservative treatment exhibited predominantly pro-inflammatory dietary patterns, as reflected by exclusively positive DII scores. In adjusted multivariate analysis, higher DII scores remained associated with higher CI values, even after controlling for age, sex, and renal function. This finding suggests a potential relationship between dietary inflammatory potential and greater central adiposity in patients with CKD. Given that central adiposity is a well-established cardiovascular risk factor, these results may indicate that more pro-inflammatory dietary patterns are linked to less favorable anthropometric cardiovascular profiles in this population. The inclusion of eGFR in the regression model strengthens the analysis by accounting for renal function, an important confounding factor in CKD populations, suggesting that the observed association between DII and CI is not solely explained by disease severity.

To our knowledge, studies evaluating the DII in Brazilian patients with CKD under conservative treatment remain scarce. The predominance of positive DII values observed in this study suggests that these individuals may follow dietary patterns with substantial inflammatory potential. This finding may reflect the persistence of overly restrictive dietary practices, particularly regarding plant-based foods, in patients who have not yet received specialized nutritional counseling, as previously described in CKD dietary management guidelines and the renal nutrition literature^1,28^. In this context, the DII may represent a useful adjunct tool for identifying dietary patterns potentially associated with cardiometabolic risk in this population.

A relevant finding of the present study was the association between higher DII scores and higher CI values, suggesting that a more pro-inflammatory diet may be linked to increased central adiposity and cardiovascular risk in this population. This result is clinically meaningful given the elevated cardiovascular burden observed in CKD patients and is consistent with previous evidence linking higher DII scores to less favorable cardiometabolic profiles in other populations^29,30,31^. Although causality cannot be inferred due to the cross-sectional design, these findings reinforce the potential utility of the DII as part of nutritional and cardiometabolic risk assessment in CKD.

The relatively preserved Castelli indices observed in the study population may be partially explained by the frequent use of lipid-lowering medications, particularly statins, which may have attenuated lipid abnormalities and potentially obscured associations between dietary inflammatory potential and lipid-derived cardiovascular risk indices.

In multivariate analysis, fiber and flavonol intake remained independently associated with lower DII scores. However, these findings should be interpreted with caution, since both variables are components of the DII calculation and are therefore inherently related to the index. Rather than representing novel associations, these results primarily reinforce the internal consistency of the DII in this population, as originally proposed in the index development and validation studies^6,27^.

This study has several limitations that should be considered when interpreting the findings. First, the relatively small sample size may have limited the statistical power to detect associations and restricted the generalizability of the results. Second, the cross-sectional design precludes establishing temporal or causal relationships between dietary inflammatory potential and clinical outcomes. Third, no inflammatory biomarkers were measured; therefore, this study assessed the inflammatory potential of the diet rather than systemic inflammation itself.

Although the DII remained significantly associated with the CI after adjustment for age, sex, and eGFR, the modest explanatory power of the regression model suggests that additional unmeasured factors likely contribute to central adiposity in this population. Furthermore, biochemical parameters were obtained from medical records within a two-month window relative to dietary assessment, which may have introduced temporal mismatch bias, particularly given the potential variability of metabolic and renal parameters in patients with CKD. Finally, dietary intake assessment based on 24-hour recalls remains subject to recall bias, underreporting, and imprecision in estimating culinary ingredients such as seasonings and added sodium, which may affect the accuracy of DII calculation.

## CONCLUSION

In conclusion, patients with CKD undergoing conservative treatment exhibited predominantly pro-inflammatory dietary patterns. Higher DII scores were associated with greater central adiposity, even after adjustment for age, sex, and renal function, suggesting a potential relationship between dietary inflammatory potential and cardiovascular risk in this population. These findings reinforce the importance of evaluating overall dietary patterns rather than isolated nutrients in CKD management. In addition, these findings support the potential utility of the DII as a complementary tool in nutritional assessment; however, prospective studies are needed to confirm the clinical relevance of these associations.

## Data Availability

The datasets generated and/or analyzed during the current study are not publicly available due to ethical, legal, and privacy restrictions but are available from the corresponding author upon reasonable request.
