# Supplementary material for: Dietary inflammatory index in patients with chronic kidney disease under conservative treatment at a university hospital in Rio de Janeiro, Brazil
Source: J Bras Nefrol. 2026 Aug 17;48(4):e20250350. doi: 10.1590/2175-8239-JBN-2025-0350en (PMC13491751; doi:10.1590/2175-8239-JBN-2025-0350en)
Supplement: Translation

# Translation: Índice inflamatório da dieta em pacientes com doença renal crônica em tratamento conservador atendidos em um hospital universitário no Rio de Janeiro, Brasil

**DOI:** 10.1590/2175-8239-JBN-2025-0350pt

**Authors:** Karine Campos Ladeira, Amália Almeida Bastos, Raquel Martins Martinez, Tatiana Pereira de Paula

**Affiliations:** 1 Universidade Federal do Rio de Janeiro, Instituto de Nutrição Josué de Castro, Programa de Pós-Graduação em Nutrição Clínica, Rio de Janeiro, RJ, Brasil.; 2 Universidade de São Paulo, Programa de Pós-Graduação em Nutrição em Saúde Pública, São Paulo, SP, Brasil.; 3 Universidade Federal do Estado do Rio de Janeiro, Programa de Pós-Graduação em Alimentação e Nutrição, Rio de Janeiro, RJ, Brasil.

**Keywords:** Insuficiência Renal Crônica, Inflamação, Dieta, Fatores de Risco de Doenças Cardíacas, Estado Nutricional.

## Abstract

**Introdução:**

A doença renal crônica (DRC) está associada a um estado inflamatório crônico de baixo grau, que pode ser influenciado pelos padrões alimentares. O Índice Inflamatório da Dieta (IID) é uma ferramenta validada utilizada para estimar o potencial inflamatório da alimentação.

**Objetivo:**

Avaliar o potencial inflamatório da dieta em pacientes com DRC em tratamento conservador e sua associação com marcadores antropométricos e de risco cardiovascular.

**Métodos:**

Estudo observacional transversal conduzido com pacientes adultos com DRC estágios 3 a 5 em tratamento conservador. O consumo alimentar foi avaliado por meio de dois recordatórios de 24 horas não consecutivos, e o IID foi calculado com base em 36 parâmetros dietéticos. Foram avaliados indicadores antropométricos e de risco cardiovascular, incluindo índice de massa corporal (IMC), dobra cutânea tricipital (DCT), área muscular do braço (AMB), relação cintura/estatura (RCE), índices de Castelli e índice de conicidade (IC). Modelos de regressão linear multivariada foram ajustados para idade, sexo e taxa de filtração glomerular estimada (TFGe).

**Resultados:**

Foram incluídos 51 pacientes, com mediana de idade de 71 anos (IIQ, 56–80), sendo 56,9% do sexo feminino. A mediana do IID foi 2,90 (IIQ, 2,29–3,74), indicando padrão alimentar predominantemente pró-inflamatório. Escores mais elevados do IID estiveram associados a maiores valores do índice de conicidade (β = 0,368; *p* = 0,010). Não foram observadas associações estatisticamente significativas entre o IID e outros marcadores antropométricos, bioquímicos ou de risco cardiovascular.

**Conclusão:**

Pacientes com DRC em tratamento conservador apresentaram padrão alimentar predominantemente pró-inflamatório. Escores mais elevados do IID estiveram associados a maior adiposidade central.

## INTRODUÇÃO

Mais de 850 milhões de pessoas em todo o mundo sofrem de algum tipo de doença renal, sendo a doença renal crônica (DRC) responsável por mais de um milhão de óbitos a cada ano. Estima-se que o envelhecimento e o crescimento populacional levarão a um aumento significativo na prevalência da DRC nas próximas décadas, especialmente em países de baixa e média renda, onde grandes parcelas da população não têm acesso ao seu diagnóstico, prevenção e tratamento^1^.

A DRC é caracterizada por uma perda progressiva e irreversível da função renal ao longo de um período mínimo de três meses. Essa redução pode ser detectada por exames de imagem, sangue e urina^1^. A progressão da doença leva à diminuição da excreção urinária de íons, eletrólitos e, principalmente, dos produtos nitrogenados provenientes do metabolismo proteico e da ingestão de proteínas. Consequentemente, ocorre acidose metabólica de baixo grau, acompanhada de complicações clínicas e nutricionais, o que aumenta o risco de morbidade e mortalidade^2,3^.

Os mecanismos da DRC geram um processo inflamatório crônico e persistente, de causas multifatoriais. Entre elas destacam-se o aumento da produção de citocinas pró-inflamatórias, o estresse oxidativo, a acidose, infecções crônicas e recorrentes, alterações no metabolismo do tecido adiposo e a disbiose intestinal. Esses fatores resultam em danos repetidos ao endotélio vascular, cicatrização e fibrose^2,3^.

O controle rigoroso dos fatores de risco modificáveis tem como objetivo reduzir a progressão da doença. Devem ser considerados: hipertensão arterial sistêmica (HAS), diabetes e controle glicêmico, dislipidemia, obesidade, tabagismo e hábitos alimentares^1,4^.

Padrões alimentares ricos em antioxidantes, compostos anti-inflamatórios e nutricionalmente equilibrados — como aqueles que enfatizam o consumo de alimentos de origem vegetal, incluindo a dieta mediterrânea, a *Dietary Approaches to Stop Hypertension* (DASH), além de dietas vegetarianas e veganas — têm demonstrado eficácia na atenuação da inflamação na DRC^5^. Além da composição nutricional, a presença de fitoquímicos confere um importante efeito anti-inflamatório. Compostos bioativos, como resveratrol, alicina, cinamaldeído, quercetina, proantocianidina, catequina, curcumina, epigalocatequina-3-galato, fisetina, sulforafano e ácidos clorogênicos, demonstraram modular a expressão de NRF2, suprimir a expressão de NLRP3 e regular características inflamatórias e pró-oxidativas em pacientes com DRC^5^.

O Índice Inflamatório da Dieta (IID) é uma ferramenta validada que apresenta boa capacidade de avaliar o potencial inflamatório da dieta. Foi desenvolvido a partir da associação entre o potencial inflamatório da alimentação e as concentrações séricas de proteína C-reativa (PCR). O IID é estimado por meio de escores ponderados baseados em evidências científicas, considerando componentes alimentares com características pró- e anti-inflamatórias. Quanto mais positiva e elevada for a pontuação, mais pró-inflamatória é a dieta, enquanto valores mais baixos e negativos estão associados a uma dieta anti-inflamatória^6^.

Alguns autores têm demonstrado uma relação importante entre o IID e a doença renal crônica. Mazidi et al.^7^ investigaram a associação entre o IID e a prevalência de DRC em adultos norte-americanos participantes do *National Health and Nutrition Examination Survey* (NHANES), com dados de função renal coletados entre 2005 e 2012, incluindo 21.649 participantes, dos quais 1.634 (6,8%) apresentavam DRC. Entre outros indicadores, os autores concluíram que uma dieta pró-inflamatória está associada à diminuição da função renal e à elevada prevalência de DRC. Em uma meta-análise, Kachouei et al.^8^ demonstraram associação positiva significativa entre o IID e a probabilidade de DRC e baixa taxa de filtração glomerular (TFG), embora os resultados sobre a correlação entre o IID e biomarcadores séricos de função renal ainda sejam inconclusivos. Assim, estudos que avaliam o uso desse índice na DRC ainda são escassos, sendo necessária uma maior produção de dados em diferentes regiões do mundo para validar essa associação.

Considerando que a DRC está associada a um estado inflamatório crônico, escores elevados de IID podem estar relacionados a desfechos clínicos desfavoráveis. Dessa forma, o objetivo primário deste estudo foi investigar a associação entre o IID e marcadores antropométricos e aterogênicos em pacientes com doença renal crônica em tratamento conservador. Os objetivos secundários incluem descrever o perfil inflamatório da dieta da população estudada e comparar características dietéticas, clínicas e sociodemográficas de acordo com as categorias de IID.

## MÉTODOS

### Desenho do estudo

Para este estudo transversal, foi considerada uma amostra de conveniência de pacientes com diagnóstico clínico de DRC em tratamento conservador ou acompanhamento no Ambulatório de Nefrologia do Hospital Universitário Clementino Fraga Filho (HUCFF/Rio de Janeiro, RJ, Brasil), sem consulta prévia no ambulatório de nutrição. Não foi realizado cálculo formal do tamanho da amostra, e o estudo deve ser considerado exploratório.

Os seguintes critérios foram utilizados para determinar a elegibilidade: idade superior a 18 anos, qualquer sexo, taxa de filtração glomerular estimada (TFGe) menor ou igual a 60 mL/min, ausência de doenças autoimunes (por exemplo, glomerulopatias), neoplasias malignas e ausência de histórico de transplante renal ou terapia dialítica prévia. Pacientes que se recusaram a participar de um segundo recordatório alimentar de 24 horas foram excluídos do estudo.

O estudo foi submetido e aprovado pelo Comitê de Ética em Pesquisa do HUCFF, sob o parecer nº 21.941-913, em conformidade com as recomendações da Resolução nº 466/12 do Conselho Nacional de Saúde. A participação no estudo foi voluntária, e todos os participantes foram capazes de ler e assinar o Termo de Consentimento Livre e Esclarecido.

### Perfil sociodemográfico

Foram coletadas informações sobre características sociodemográficas (idade, escolaridade, renda *per capita*, raça), duração do tratamento no HUCFF, prática de exercícios físicos, tabagismo, consumo de álcool, exames bioquímicos, medidas antropométricas e dados relativos ao consumo alimentar. Informações sobre doenças crônicas e uso de medicamentos foram obtidas por meio de autorrelato estruturado durante entrevistas presenciais conduzidas pela pesquisadora e, posteriormente, confirmadas por meio da revisão dos prontuários médicos dos pacientes. Todos os dados foram coletados por uma única pesquisadora, nutricionista.

Fumantes atuais foram definidos como aqueles que fuma­ram 100 ou mais cigarros durante a vida e que continuam fumando, enquanto ex-fumantes foram definidos como aqueles que fumaram pelo menos 100 cigarros durante a vida, mas pararam de fumar, independentemente de há quanto tempo^9^. Consumidores de bebidas alcoólicas foram definidos como aqueles que consumiram quatro ou mais doses para mulheres e cinco ou mais doses para homens em uma única ocasião nos últimos 30 dias. Uma dose é definida como uma medida padrão de bebida destilada, uma taça de vinho ou uma lata de cerveja. Ex-etilistas foram definidos como aqueles que consumiram álcool no passado, mas não beberam no ano corrente^4^. O nível de escolaridade foi avaliado em dois grupos: baixa escolaridade (ensino fundamental incompleto ou completo, ou ensino médio incompleto) e alta escolaridade (ensino médio completo, ensino superior incompleto ou completo). A classificação da renda familiar *per capita* foi determinada com base na comparação com o salário mínimo brasileiro de 2023, sendo os níveis de renda categorizados como iguais ou inferiores a um salário mínimo ou superiores a um salário mínimo. Em relação ao fator atividade física, os indivíduos foram considerados ativos se praticassem, no mínimo, 150 a 300 minutos de atividade física aeróbica de intensidade moderada, ou, no mínimo, 75 a 150 minutos de atividade física aeróbica de intensidade vigorosa, ou uma combinação equivalente de atividades físicas de intensidade moderada e vigorosa ao longo da semana. As atividades aeróbicas foram categorizadas como atividades de resistência que melhoram o condicionamento cardiorrespiratório, incluindo caminhada, corrida, natação e ciclismo^10^.

### Perfil nutricional

Medidas antropométricas de massa corporal, estatura, circunferência do braço (CB), perímetro da cintura (CC) e dobra cutânea tricipital (DCT) foram coletadas de acordo com protocolos estabelecidos^11,12^. A massa corporal foi medida utilizando uma balança digital (FILIZOLA® PL 180) com capacidade máxima de 150 kg e precisão de 0,1 kg. A estatura dos participantes foi medida em metros, utilizando um estadiômetro de parede (TONELLI®) com precisão de 0,1 cm. As medidas de perímetros e dobras cutâneas foram obtidas utilizando os seguintes instrumentos: uma fita métrica antropométrica flexível de aço e um adipômetro científico (CESCORF®).

A estimativa do índice de massa corporal (IMC) foi baseada nas medidas de peso e estatura. O diagnóstico nutricional foi baseado nos valores do IMC: peso normal (IMC = 18,5–24,9 kg/m^2^), sobrepeso (IMC = 25–29,9 kg/m^2^), obesidade grau I (IMC = 30–34,9 kg/m^2^) e obesidade grau II (IMC = 35–39,9 kg/m^2^). No caso de idosos, foram consideradas as seguintes categorias de IMC: baixo peso (IMC < 23 kg/m^2^), peso adequado (23 < IMC < 28 kg/m^2^), sobrepeso (28 < IMC < 30 kg/m^2^) e obesidade (IMC > 30 kg/m^2^)^12,13^. Para avaliar a massa muscular, a área muscular do braço (AMB) foi calculada da seguinte forma: AMB = [CB – (DCT × π)^2^] ÷ (4 × π)^12^. Os valores foram comparados aos recomendados por Frisancho^14^, de acordo com idade e sexo, sendo os indivíduos com percentis abaixo de 15 classificados como apresentando depleção moderada de massa magra e aqueles com percentis abaixo de 5, como apresentando depleção grave.

### Perfil bioquímico e função renal

Os resultados dos exames laboratoriais foram obtidos do sistema informatizado de atendimento ao paciente do HUCFF, com data de até dois meses antes da coleta de dados. Os parâmetros séricos analisados foram: ureia, creatinina, glicose, hemoglobina glicada (HbA1c), hemoglobina, albumina, colesterol total, colesterol de lipoproteína de alta densidade (HDL-c), colesterol de lipoproteína de baixa densidade (LDL-c), triglicerídeos, fósforo, potássio, cálcio, hormônio da paratireoide (PTH) e vitamina D. A função renal foi avaliada pelo cálculo da taxa de filtração glomerular estimada (TFGe), utilizando a equação da CKD-*Epidemiology Collaboration* (CKD-EPI), recomendada pelas diretrizes da *National Kidney Foundation* para a *Kidney Disease Outcomes Quality Initiative* ^1^.

### Indicadores de adiposidade abdominal e risco aterogênico

A adiposidade central foi avaliada pela relação cintura/estatura (RCE), adotando-se o ponto de corte de 0,55, conforme determinado por Lin et al.^15^, para indicar elevada adiposidade central. A avaliação da gordura abdominal foi realizada por meio do cálculo do índice de conicidade (IC), determinado pela seguinte equação matemática: IC = CC (m)/[0,109 × (√P(kg)/A(m))]^16^. Para a classificação do risco cardiovascular, foram utilizados os valores propostos por Pitanga e Lessa^17^, correspondentes a valores ≥ 1,18 para mulheres e ≥ 1,25 para homens.

Os índices de Castelli I (relação entre colesterol total e HDL-c) e II (relação entre LDL-c e HDL-c) foram utilizados para a avaliação do risco aterogênico. Foram adotados como valores de referência: índice de Castelli I < 4,0 para mulheres e < 5,0 para homens, e índice de Castelli II < 3,0 para mulheres e < 3,5 para homens^18,19^.

### Consumo alimentar

O consumo alimentar foi avaliado por meio do recordatório de 24 horas (R24h), utilizando o método de múltiplas passagens validado por Conway et al.^20^, que consiste em cinco etapas: (1) listagem ininterrupta dos alimentos e bebidas consumidos; (2) investigação de grupos alimentares frequentemente consumidos; (3) registro do horário e da ocasião de consumo; (4) detalhamento das quantidades e da descrição dos alimentos ingeridos, com auxílio de um álbum fotográfico; e (5) revisão final das informações fornecidas. Foram aplicados dois recordatórios em dias distintos e não consecutivos, com o objetivo de contemplar adequadamente dias úteis e finais de semana na população estudada. A primeira coleta foi realizada presencialmente, enquanto a segunda foi obtida por contato telefônico.

As porções consumidas foram descritas em medidas caseiras e posteriormente convertidas em massa e volume utilizando a Tabela para Avaliação de Consumo Alimentar em Medidas Caseiras^21^. A estimativa de macronutrientes, minerais e energia foi realizada com base na Tabela Brasileira de Composição de Alimentos^22^. O conteúdo de flavonoides e carotenoides foi determinado a partir de tabelas complementares da mesma base de dados^23,24^. Também foi utilizada a base de dados *Phenol Explorer* ^25^. Os valores de cafeína dos alimentos seguiram as estimativas propostas por Rocha et al.^26^. As quantidades de alho e cebola foram estimadas para cada 100 g de preparação: para arroz e carnes, considerou-se 1 g de alho e 4 g de cebola; para feijão, 0,2 g de alho e 4 g de cebola. Para temperos, a cada 100 g de preparação foram considerados, quando utilizados, 0,35 g de orégano, 0,74 g de pimenta-do-reino e 0,4 g de alecrim.

Os dados sobre nutrientes e alimentos provenientes dos dois inquéritos de recordatório foram utilizados para estimar a ingestão habitual de cada indivíduo. A distribuição do consumo foi corrigida em função da variabilidade intraindividual por meio do programa *Multiple Source Method* (MSM), que calcula a ingestão individual de cada nutriente e alimento analisado.

### Índice inflamatório da dieta (IID)

O método descrito por Shivappa et al.^27^ foi utilizado para o cálculo do IID, considerando a variação entre componentes pró-inflamatórios (com escores positivos) e anti-inflamatórios (com escores negativos). Para a obtenção do escore do IID, foram considerados 36 parâmetros dietéticos: ácido fólico, ácidos graxos monoinsaturados, ácidos graxos poli-insaturados, β-caroteno, carboidratos, proteínas, colesterol, energia, fibras, flavonóis, flavonas, flavanonas, flavan-3-ol, antocianidinas, gordura saturada, gordura total, gordura trans, vitaminas B1, B2, B3, B6, B12, A, D, C e E, magnésio, zinco, ferro, selênio, alho, cebola, orégano, pimenta, alecrim e cafeína. Os parâmetros eugenol, álcool, gengibre, cúrcuma, chá verde e chá preto foram excluídos do cálculo do IID, uma vez que não foi relatado qualquer consumo desses itens na população do estudo. Esta abordagem está em consonância com estudos anteriores que aplicaram o IID na ausência de dados alimentares completos^27^. A presença de isoflavonas e cúrcuma foi observada na dieta de apenas um paciente e, portanto, esses alimentos não foram considerados de consumo habitual pela população do estudo.

A pontuação de cada parâmetro foi calculada e, em seguida, somada para obter uma pontuação de IID individual. Posteriormente, as pontuações de IID calculadas para todos os parâmetros alimentares foram somadas para obter a pontuação total do IID de cada indivíduo. Para minimizar a diferença na ingestão energética entre os indivíduos, a pontuação do IID foi ajustada em função da energia, convertendo todos os alimentos para um consumo de 1.000 kcal.

### Análise estatística

As análises estatísticas foram realizadas utilizando o software *Statistical Package for the Social Sciences*® (SPSS®, versão 22 para Windows; SPSS Inc., Chicago, IL, EUA). A distribuição das variáveis foi avaliada pelo teste de Shapiro-Wilk. Variáveis contínuas com distribuição normal foram descritas como média e desvio padrão (DP), enquanto aquelas com distribuição não normal foram descritas como mediana e intervalo inter­quartil (IIQ). Variáveis categóricas foram apresentadas como frequências absolutas (n) e relativas (%).

A categorização do IID com base na mediana foi utilizada apenas para comparações descritivas exploratórias, enquanto o IID foi mantido como variável contínua nas análises de regressão. A multicolinearidade foi avaliada por meio da análise de correlação entre variáveis independentes, sendo incluídas nos modelos apenas aquelas não intrinsecamente relacionadas ao cálculo do IID.

Para avaliar a associação entre o potencial inflamatório da dieta e marcadores antropométricos de risco cardiovascular, foi realizada regressão linear multivariada utilizando o IC contínuo como variável dependente e o escore do IID como principal variável independente. O modelo foi ajustado para idade, sexo e taxa de filtração glomerular estimada (TFGe). O nível de significância estatística adotado foi de *p* < 0,05.

## RESULTADOS

Do total de 61 pacientes com DRC, 10 foram excluídos devido à ausência de dados de consumo alimentar. As características sociodemográficas e de estilo de vida, estratificadas de acordo com as categorias de IID (baixo e elevado), são apresentadas na Tabela 1. Não foram observadas diferenças significativas entre os grupos de IID no que diz respeito à idade, sexo, atividade física, tabagismo, consumo de álcool, diagnóstico primário de DRC ou renda (*p* > 0,05), enquanto o nível de escolaridade diferiu significativamente entre os grupos (*p* = 0,039).

**Tabela 1:** Características sociodemográficas de pacientes com DRC submetidos a tratamento conservador em hospital universitário no Rio de Janeiro, Brasil, estratificadas por pontuação IID, com base na mediana observada para essa pontuação nesta população.

Características	(IID < 2,9029)	(IID > 2,9029)	*p*
**Idade (anos)**			
*Mediana (IIQ)*	70 (39–88)	71,5 (31–86)	0,116
**Sexo**			
Masculino	52% (n = 13)	65,4% (n = 17)	0,157
Feminino	48% (n = 12)	34,6% (n = 9)
**Atividade física**			
Ativo	8% (n = 2)	0% (n = 0)	0,216
Inativo	92% (n = 23)	100% (n = 26)
**Tabagismo**			
Nunca fumou	82,4% (n = 20)	82,4% (n = 22)	0,187
Ex-fumante	17,6% (n = 5)	17,6% (n = 4)
Fumante	0% (n = 0)	0% (n = 0)
**Consumo de álcool**			
Não consumo	86,3% (n = 20)	92,3% (n = 24)
Ex-etilista	13,7% (n = 5)	7,7% (n = 2)	0,163
Etilista	0% (n = 0)	0% (n = 0)
**Escolaridade**			
Baixa escolaridade	66,7% (n = 17)	65,4% (n = 17)	0,039
Alta escolaridade	33,3% (n = 8)	34,6% (n = 9)
**Diagnóstico primário de DRC**			
Hipertensão	72% (n = 18)	53,8% (n = 14)	0,086
Diabetes tipo 2	12% (n = 3)	15,4% (n = 4)
Outras causas	16% (n = 4)	30,8% (n = 8)
**Renda *per capita* (salário mínimo)**			
≤ 1 salário mínimo	68% (n = 17)	73,1% (n = 19)	0,688
> 1 salário mínimo	32% (n = 8)	26,9% (n = 7)

Notas – Idade apresentada como mediana e intervalo interquartil (IIQ); todas as demais variáveis foram apresentadas como frequência (%). Atividade física definida como a prática de no mínimo 150–300 minutos de atividade física aeróbica de intensidade moderada, ou de no mínimo 75–150 minutos de atividade física aeróbica de intensidade vigorosa, ou uma combinação equivalente de atividades físicas de intensidade moderada e vigorosa ao longo da semana. Ex-fumantes definidos como indivíduos que fumaram pelo menos 100 cigarros ao longo da vida, mas cessaram o tabagismo. Ex-etilistas definidos como indivíduos que consumiram álcool no passado, mas não consumiram álcool durante o ano corrente. Alta escolaridade definido como ensino médio completo, ensino superior incompleto ou ensino superior completo. Renda familiar classificada de acordo com o salário mínimo brasileiro vigente no ano de 2023.

Os pacientes selecionados referiram utilizar medicamentos anti-hipertensivos, com uma proporção notável (n = 34) de uso de medicamentos hipolipemiantes, 41,18% (n = 21) de uso de medicamentos hipoglicemiantes e 15,69% (n = 8) de uso de colecalciferol. De acordo com o diagnóstico primário de DRC, 32 indivíduos apresentavam HAS (62,7%), seguidos de diabetes mellitus tipo 2 (13,7%), doença renal policística (7,8%), nefrolitíase (5,9%), infecção do trato urinário (3,9%), pielonefrite (2%), rim único (2%) e diabetes mellitus tipo 1 (2%). O tempo de acompanhamento da DRC na clínica de Nefrologia do HUCFF foi de 2,67 ± 1,7 anos.

Em relação aos parâmetros bioquímicos, os valores de albumina (4 ± 0,56 g/dL), triglicerídeos (136 ± 69,27 mg/dL), colesterol total (180 mg/dL; IIQ, 151–203), LDL-c (101,7 ± 41,52 mg/dL), potássio (4,82 ± 0,62 mEq/L), fósforo (4,04 ± 0,82 mg/dL) e cálcio (9,24 ± 0,63 mg/dL) encontravam-se dentro da faixa de normalidade. A mediana da HbA1c estava elevada (6,3% (5,6–7,7)). Os demais parâmetros alterados foram compatíveis com a presença de DRC.

As associações entre o IID e os componentes dietéticos incluídos no cálculo do índice estão apresentadas na Tabela 2.

**Tabela 2:** Comparação da ingestão de nutrientes entre pacientes com DRC estratificados pela mediana do IID, atendidos em um hospital universitário do Rio de Janeiro, Brasil.

Parâmetro	(IID < 2,9029)	(IID > 2,9029)	Valor de *p*
Calorias (kcal)	1512,66 ± 197,851	1567,17 ± 306,48	0,456
Proteína (g)	78,47 ± 9,66	74,41 ± 14,76	0,254
Carboidratos (g)	212,17 ± 31,98	198,48 ± 33,69	0,143
Lipídeos (g)	48,16 ± 4,69	51,22 ± 7,14	0,790
Fibras (g)	31,06 ± 5,17	21,67 ± 6,51	0,000
Colesterol (mg)	274,67 ± 89,30	268,69 ± 88,22	0,811
Gordura saturada (g)	19,56 ± 0,40	19,40 ± 0,437	0,185
Gordura monoinsaturada (g)	14,54 ± 1,83	15,63 ± 2,67	0,094
Gordura poli-insaturada (g)	6,88 ± 2,21	8,71 ± 2,74	0,011
Gordura trans (g)	1,22 ± 0,44	1,57 ± 0,57	0,019
Cálcio (mg)	645,13 ± 109,13	615,69 ± 155,93	0,441
Ferro (mg)	11,01 ± 1,56	10,48 ± 2,39	0,355
Magnésio (mg)	257,56 ± 35,28	219,25 ± 39,35	0,001
Zinco (mg)	11,48 ± 1,56	10,35 ± 3,04	0,106
Flavonóis (mg)	0,55 (0,10–3,40)	0,40 (0,05–1,30)	0,297
Alho (g)	4,20 (2,95–4,20)	3,20 (2,20–4,20)	0,075
Cebola (g)	20,00 (12,10–20,00)	16,00 (12,10–20,00)	0,160
Cafeína (mg)	75,15 (10,57–137,50)	67,71 (11,60–116,50)	0,901
β-caroteno (µg)	699,60 (39,4–2577,5)	442,20 (253,90–442,20)	0,304
Pimenta-do-reino (g)	0,40 (0,35–0,60)	0,40 (0,20–0,55)	0,603
Selênio (mcg)	40,65 (30,67–50,55)	38,30 (30,25–46,60)	0,977
Vitamina A RE (mcg)	389,53 ± 85,18	386,41 ± 82,14	0,895
Vitamina D (mcg)	1,25 (0,45–2,32)	1,70 (0,20–3,25)	0,962
Vitamina C (mg)	108,80 (52,80–150,50)	49,80 (33,90–85,150)	0,001
Vitamina B1 (mg)	1,03 ± 0,16	1,03 ± 0,27	0,984
Vitamina B2 (mg)	1,50 ± 0,46	1,60 ± 0,55	0,483
Vitamina B3 (mg)	20,30 ± 4,80	20,94 ± 5,67	0,665
Vitamina B6 (mg)	0,80 (0,50–1,00)	0,80 (0,60–1,00)	0,614
Vitamina B12 (mcg)	3,20 (2,70–4,20)	3,20 (2,40–4,90)	0,977
Vitamina E (mg)	4,53 ± 0,78	4,62 ± 0,85	0,697
Vitamina B9 (mcg)	432,15 (399,20–488,00)	348,00 (290,50–413,30)	0,000
Sódio (mg)	1432,77 ± 200,33	1596,95 ± 260,16	0,015
Cobre (mg)	1,81 ± 0,33	1,33 ± 0,41	0,000
Fósforo (mg)	1147,21 ± 177,80	1094,77 ± 216,73	0,349
Potássio (mg)	2465,36 ± 278,35	2215,44 ± 298,08	0,003

Abreviatura – Vitamina A RE: Vitamina A equivalente de retinol.

A Tabela 3 apresenta o perfil nutricional e os fatores de risco cardiovascular dos pacientes com doença renal crônica estratificados de acordo com a mediana do IID. Entre os adultos, observou-se predominância de indivíduos eutróficos em ambos os grupos de IID, enquanto, entre os idosos, houve maior frequência de excesso de peso, especialmente no grupo com IID mais elevado. Em relação à AMB, a maioria dos pacientes apresentou valores dentro da faixa de adequação (percentil ≥ 16 e ≤ 85) em ambos os grupos.

**Tabela 3:** Perfil nutricional e fatores de risco cardiovascular de pacientes com DRC atendidos em um hospital universitário no Rio de Janeiro, Brasil, estratificados por pontuação IID, com base na pontuação mediana observada nesta população.

Parâmetros	(IID < 2,9029)	(IID > 2,9029)	*p*
**IMC – adultos (n = 15)**			
< 18,50 kg/m^2^	0% (n = 0)	6,67% (n = 1)	0,645
≥ 18,50 kg/m^2^ – ≤ 24,90 kg/m^2^	26,67% (n = 4)	26,67% (n = 4)
≥ 25,00 kg/m^2^ – ≤ 29,90 kg/m^2^	6,67% (n = 1)	6,67% (n = 1)
≥ 30,00 kg/m^2^ – ≤ 34,90 kg/m^2^	0% (n = 0)	13,33% (n = 2)
≥ 34,90 kg/m^2^ – ≤ 39,90 kg/m^2^	6,67% (n = 1)	6,67% (n = 1)
**IMC – idosos (n = 36)**			
< 23,00 kg/m^2^	16,67% (n = 6)	8,33% (n = 3)	0,619
≥ 23,00 kg/m^2^ – ≤ 28,00 kg/m^2^	16,67% (n = 6)	16,67% (n = 6)
> 28,00 kg/m^2^	19,44% (n = 7)	22,22% (n = 8)
**AMB (n = 51)**			
Percentil < 5	3,92% (n = 2)	0% (n = 0)	0,453
Percentil ≥ 5 – ≤ 15	5,88% (n = 3)	7,84% (n = 4)
Percentil ≥ 16 – ≤ 85	33,33% (n = 17)	33,33% (n = 17)
Percentil ≥ 95	5,88% (n = 3)	9,80% (n = 5)
**RCE (n = 51)**			
≤ 0,55	29,41% (n = 15)	19,61% (n = 10)	0,473
≥ 0,55	21,57% (n = 11)	29,41% (n = 15)
**IC – feminino (n = 29)**			
≤ 1,18	20,69% (n = 6)	20,69% (n = 6)	0,341
≥ 1,18	20,69% (n = 6)	37,93% (n = 11)
**IC – masculino (n = 22)**			
≤ 1,25	36,36% (n = 8)	22,73% (n = 5)	0,082
≥ 1,25	9,09% (n = 2)	31,82% (n = 7)
**Índice de Castelli I – feminino (n = 28)**			
≤ 4,00	25,00% (n = 7)	17,86% (n = 5)	0,299
≥ 4,00	42,86% (n = 12)	14,29% (n = 4)
**Índice de Castelli I – masculino (n = 18)**			
≤ 5,00	50,00% (n = 9)	5,56% (n = 1)	0,556
≥ 5,00	44,44% (n = 8)	0% (n = 0)
**Índice de Castelli II – feminino (n = 25)**			
≤ 3,00	36,00% (n = 9)	8,00% (n = 2)	0,305
≥ 3,00	36,00% (n = 9)	20,00% (n = 5)
**Índice de Castelli II – masculino (n = 21)**			
≤ 3,50	52,38% (n = 11)	0% (n = 0)	0,476
≥ 3,50	42,86% (n = 9)	4,76% (n = 1)

Abreviaturas – IMC: índice de massa corporal; AMB: área muscular do braço; RCE: relação cintura/estatura; IC: índice de conicidade.

Quanto aos indicadores de adiposidade central, indivíduos com IID mais elevado apresentaram maior frequência de RCE aumentada e maiores proporções de IC elevado, tanto em mulheres quanto em homens. Entre as mulheres, 37,93% das participantes com IID acima da mediana apresentaram IC elevado, enquanto, entre os homens, essa frequência foi de 31,82%.

Para os índices aterogênicos, observou-se predominância de valores elevados do índice de Castelli I entre as mulheres com IID abaixo da mediana. Já entre os homens, a maior parte apresentou valores normais para os índices de Castelli I e II. Não foram observadas diferenças estatisticamente significativas entre os grupos para as variáveis avaliadas.

No contexto da avaliação de risco cardiovascular, a Tabela 4 demonstra correlação positiva entre o IID e o IC (r = 0,333; p = 0,017), indicando associação entre maior potencial inflamatório da dieta e maior adiposidade central. Não foram observadas correlações estatisticamente significativas entre o IID e os demais marcadores de risco cardiovascular avaliados.

**Tabela 4:** Correlação entre o IID e as variáveis de risco cardiovascular em pacientes com DRC atendidos em um hospital universitário no Rio de Janeiro, Brasil.

Risco cardiovascular	*r*	Valor de *p*
IC	0,333	0,017
Índice de Castelli I	0,239	0,091
Índice de Castelli II	0,009	0,949
RCE	0,247	0,081

Abreviaturas – RCE: relação cintura/estatura; IC: índice de conicidade; *r*: coeficiente de correlação.

Na análise de regressão linear multivariada, utilizando o IC contínuo como variável dependente e ajustada para idade, sexo e TFGe, escores mais elevados do IID estiveram positivamente associados a maiores valores do IC (*B* = 0,043, β padronizado = 0,368, *p* = 0,010) (Tabela 5). Não foram observadas associações estatisticamente significativas para idade, sexo ou TFGe.

**Tabela 5:** Análise de regressão linear multivariada dos fatores associados ao índice de conicidade contínuo observado em pacientes com DRC atendidos em um hospital universitário no Rio de Janeiro, Brasil.

Variáveis	Efeitos ajustados			
*B*	β padronizado	*t*	Valor de *p*
Escore do IID	0,043	0,368	2,669	0,010
Idade (anos)	0,001	0,143	0,892	0,377
Sexo	0,000	0,001	0,008	0,993
TFGe	-0,001	-0,106	-0,668	0,508

Notas – Modelo ajustado para idade, sexo e TFGe, com apresentação de coeficientes não padronizados (*B*) e coeficientes padronizados (β). Nível de significância estatística estabelecido em *p* < 0,05. Ajuste do modelo: R² = 0,156; R² ajustado = 0,083; *F* = 2,129; *p* = 0,092.

## DISCUSSÃO

O presente estudo demonstrou que pacientes com DRC em tratamento conservador apresentaram padrões alimentares predominantemente pró-inflamatórios, refletidos por escores de IID exclusivamente positivos. Na análise multivariada ajustada, escores mais elevados do IID permaneceram associados a maiores valores do IC, mesmo após o controle para idade, sexo e função renal. Esse achado sugere uma possível relação entre o potencial inflamatório da dieta e maior adiposidade central em pacientes com DRC. Considerando que a adiposidade central é um fator de risco cardiovascular bem estabelecido, esses resultados podem indicar que padrões alimentares mais pró-inflamatórios estão associados a perfis antropométricos cardiovasculares menos favoráveis nessa população. A inclusão da TFGe no modelo de regressão fortalece a análise ao considerar a função renal um importante fator de confusão em populações com DRC, sugerindo que a associação observada entre IID e IC não é explicada apenas pela gravidade da doença.

Até onde sabemos, estudos que avaliam o IID em pacientes brasileiros com DRC submetidos a tratamento conservador ainda são escassos. A predominância de valores positivos do IID observada neste estudo sugere que esses indivíduos podem seguir padrões alimentares com elevado potencial inflamatório. Esse achado pode refletir a persistência de práticas alimentares excessivamente restritivas, especialmente em relação aos alimentos de origem vegetal, em pacientes que ainda não receberam orientação nutricional especializada, conforme previamente descrito nas diretrizes de manejo dietético da DRC e na literatura sobre nutrição renal^1,28^. Nesse contexto, o IID pode representar uma ferramenta complementar útil para identificar padrões alimentares potencialmente associados ao risco cardiometabólico nessa população.

Um achado relevante do presente estudo foi a associação entre escores mais elevados de IID e maiores valores de IC, sugerindo que uma dieta mais pró-inflamatória pode estar associada a maior adiposidade central e a maior risco cardiovascular nessa população. Esse resultado é clinicamente relevante, considerando a elevada carga de doença cardiovascular em pacientes com DRC, e é consistente com evidências prévias que associam maiores escores do IID a perfis cardiometabólicos menos favoráveis em outras populações^29,30,31^. Embora não seja possível inferir causalidade devido ao delineamento transversal, esses achados reforçam a potencial utilidade do IID como parte da avaliação nutricional e na estratificação do risco cardiometabólico na DRC.

Os índices de Castelli relativamente preservados observados na população estudada podem ser parcialmente explicados pelo uso frequente de medicamentos hipolipemiantes, especialmente estatinas, que podem ter atenuado alterações lipídicas e, possivelmente, mascarado associações entre o potencial inflamatório da dieta e os índices de risco cardiovascular derivados de lipídios.

Na análise multivariada, a ingestão de fibras e flavonóis permaneceu independentemente associada a menores escores do IID. No entanto, esses achados devem ser interpretados com cautela, uma vez que ambas as variáveis são componentes do cálculo do IID e, portanto, intrinsecamente relacionadas ao índice. Em vez de representar associações novas, esses resultados reforçam principalmente a consistência interna do IID nessa população, conforme proposto nos estudos de desenvolvimento e validação do índice^6,27^.

Este estudo apresenta algumas limitações que devem ser consideradas na interpretação dos resultados. Primeiramente, o tamanho amostral relativamente pequeno pode ter limitado o poder estatístico para detectar associações e restringido a generalização dos achados. Em segundo lugar, o delineamento transversal impede o estabelecimento de relações temporais ou causais entre o potencial inflamatório da dieta e desfechos clínicos. Em terceiro lugar, não foram avaliados biomarcadores inflamatórios; portanto, este estudo avaliou o potencial inflamatório da dieta e não a inflamação sistêmica propriamente dita.

Embora o IID tenha permanecido significativamente associado ao IC após ajuste para idade, sexo e TFGe, o poder explicativo modesto do modelo de regressão sugere que outros fatores não mensurados provavelmente contribuem para a adiposidade central nessa população. Além disso, os parâmetros bioquímicos foram obtidos a partir de prontuários médicos dentro de uma janela de dois meses em relação à avaliação dietética, o que pode ter introduzido viés de desencontro temporal, especialmente considerando a possível variabilidade dos parâmetros metabólicos e renais em pacientes com DRC. Por fim, a avaliação do consumo alimentar baseada em recordatórios de 24 horas está sujeita a viés de memória, sub-relato e imprecisão na estimativa de ingredientes culinários, como temperos e sódio adicionado, o que pode afetar a acurácia do cálculo do IID.

## CONCLUSÃO

Em conclusão, pacientes com DRC em tratamento conservador apresentaram padrões alimentares predominantemente pró-inflamatórios. Escores mais elevados do IID estiveram associados a maior adiposidade central, mesmo após ajuste para idade, sexo e função renal, sugerindo uma possível relação entre o potencial inflamatório da dieta e o risco cardiovascular nessa população. Esses achados reforçam a importância de avaliar padrões alimentares globais, em vez de nutrientes isolados, no manejo da DRC. Além disso, os resultados sugerem o uso potencial do IID como ferramenta complementar na avaliação nutricional; no entanto, estudos prospectivos são necessários para confirmar a relevância clínica dessas associações.

## Data Availability

Os conjuntos de dados gerados e/ou analisados durante o presente estudo não estão disponíveis publicamente devido a restrições éticas, legais e de privacidade, mas estão disponíveis mediante solicitação razoável ao autor correspondente.
